# The Impact of Prenatal Organophosphate Pesticide Exposures on Thai Infant Neurodevelopment

**DOI:** 10.3390/ijerph14060570

**Published:** 2017-05-27

**Authors:** Pornpimol Kongtip, Benyachalee Techasaensiri, Noppanun Nankongnab, Jane Adams, Akkarat Phamonphon, Anu Surach, Supha Sangprasert, Aree Thongsuksai, Prayoon Srikumpol, Susan Woskie

**Affiliations:** 1 Department of Occupational Health and Safety, Faculty of Public Health, Mahidol University, 420/1 Rajvithi Road, Bangkok 10400, Thailand; noppanun.nan@mahidol.ac.th (N.N.); Akkarat_ph@hotmail.com (A.P.); anu98@windowslive.com (A.S.); 2 Department of Pediatrics, Faculty of Medicine, Ramathibodi Hospital, Mahidol University, Bangkok 10400, Thailand; benyachalee@yahoo.com; 3 Department of Psychology, University of Massachusetts Boston, 100 Morrissey Blvd, Boston, MA 02125, USA; Jane.adams@umb.edu; 4 Sawanpracharak Hospital, 43 Autakavee Road, Paknumpo, Muang, Nakhon Sawan 60000, Thailand; supha135790@gmail.com; 5 Paholpolpayuhasena Hospital, 572 Sangchuto Road, Ban Tai, Kanchanaburi 71000, Thailand; areethongsuksai@gmail.com; 6 Amnatcharoen Hospital, 291 Arunprasert Road, Muang, Amnatcharoen 37000, Thailand; Prayoon1975@hotmail.com; 7 College of Health Sciences, University of Massachusetts Lowell, One University Ave, Lowell, MA 01854-2867, USA; Susan_Woskie@uml.edu

**Keywords:** organophosphate metabolites, pregnant women, infant neurodevelopment, Bayley Scales of Infant and Toddler Development

## Abstract

A birth cohort was begun to investigate the levels and sources of pesticide exposure in pregnant women living in Thailand, and to examine the effects of pesticide exposure on infant neurodevelopment at five months of age. Subjects were interviewed using questionnaires regarding their demographic characteristics, educational background, and work and home activities related to pesticide exposures. Spot urine samples were collected at 28 weeks gestation and analyzed by gas chromatography-mass spectrometry to determine maternal metabolite levels of organophosphate pesticides including dimethyl phosphate (DMP); total DEP (diethyl phosphate (DEP), diethyl thiophosphate (DETP), and diethyl dithiophosphate (DEDTP), and total DAP (the sum of all metabolite levels). At five months of age, infant development was evaluated using the Bayley Scales of Infant and Toddler Development-III (Bayley-III). Higher total DEP and total DAP metabolite levels from the mother at 28 weeks’ gestation were significantly associated with reduced motor composite scores on the Bayley-III at five months of age. The total DEP levels were also significantly associated with reduced cognitive composite scores. Prenatal concentrations of maternal urinary metabolites were associated with infant cognitive and motor development. The results of several studies now suggest the need for public health intervention to reduce prenatal pesticide exposures from both agricultural and domestic use.

## 1. Introduction

Thailand is an agricultural nation, in which approximately 38% of the population engages in agricultural work [1] and in 2014, 134,377 tons of pesticides were imported for use. The predominant category of imported pesticides was herbicides, followed by insecticides, fungicides, and others [2]. In 2012, pesticide poisonings in Thailand were reported at a rate of 12.37 per 100,000 population. Pesticide poisonings are reported most commonly during the May to August rainy season when pesticides are widely used [3]. Organophosphate pesticides are commonly used in Thailand and exposure represents a major risk during pregnancy. Median non-creatinine-adjusted urinary dialkyl phosphate (DAP) metabolite levels of 160.9 and 190.8 nmol/L were found at high levels at 28 weeks of pregnancy and at delivery period, respectively [4]. These levels are higher than those reported in pregnant women who live in an agricultural community at 106.8 nmol/L for DAP over 26 weeks according to the Center for the Health Assessment of Mothers and Children of Salinas (CHAMACOS) Study [5].

Animal studies during the 1990s clearly demonstrated the developmental neurotoxicity of organophosphate pesticides [6,7] involved in brain cell replication and differentiation, synaptic development and function, and ultimately behavioral performance. Research has now demonstrated that prenatal and early developmental exposure to organophosphate pesticides is associated with a dose-related alteration in neurobehavioral development in infancy and childhood [8,9,10,11]. The impacts on different behavioral domains—such as sensory, motor, and cognitive functions—are believed to be the result of effects on different brain areas during critical periods of neonatal development [12]. It has also been shown that neonatal exposures are likely since organophosphate pesticides have been detected in amniotic fluid [13], and umbilical cord blood [14]. The CHAMACOS study interviewed and collected urine samples from pregnant women at 14 and 27 weeks of gestation and then shortly after birth and at 6, 12, and 24 months post-natal in the expanded cohort [15]. No significant relationships were found between the average urinary organophosphate levels during pregnancy and scores on the Bayley psychomotor development index (PDI) or the mental development index (MDI) at 6 and 12 months. However, at 24 months of age, total DAP average pregnancy levels were significantly associated with lower MDI scores. [15]. The geometric mean pregnancy total DAP levels in this sample of women was 114.9 nmol/L. Investigators for the Mount Sinai Children’s Environmental Health Cohort Study [16] reported a significant negative association between increasing maternal total urinary DAP during the 26–28th week of pregnancy and Bayley MDI scores at 12 months of age in children born to Black and Hispanic women [16]. Rauh et al. [17] examined the association between cord blood levels of chlorpyrifos and infant performance on the Bayley Scale at 12, 24, and 36 months and scores on the Child Behavioral Checklist at 36 months of age. Reduced MDI and PDI scores among children at three years of age were found when comparing higher versus lower cord blood levels of chlorpyrifos [17]. Follow-ups with children at older ages have shown continued effects on general mental ability at higher exposure levels [17,18]. Reduced overall functioning appears to represent reduced performance across multiple domains: working memory [18,19]; verbal comprehension, perceptual reasoning, and processing speed [18] and attention [20].

Given these reports of adverse effects of prenatal exposures to organophosphate pesticides upon children’s neurobehavioral development, we established a birth cohort to examine these effects among the children of agricultural workers in Thailand. Both widespread exposure in the agricultural areas and the nature of the national health care system in Thailand facilitated access to exposed pregnant women and their children at regular intervals of development. We employed prospective-ascertainment and a longitudinal design to examine maternal pesticide exposures during pregnancy and offspring behavioral development at neonatal, infant, and early childhood ages. Herein, we present our findings regarding exposure characteristics and the relationship between gestational exposure and infant performance on the Bayley Scales of Infant and Toddler Development-III [21] at five months of age.

## 2. Materials and Methods

### 2.1. Study Location

This study was conducted in three geographical locations in Thailand: Amnatcharoen Hospital in Amnatcharoen province, Northeastern Thailand; Sawanpracharak Hospital in Nakhon Sawan province, in lower North Thailand; and Paholpolpayuhasena Hospital in Kanchanaburi, West Thailand. The Amnatchareon and Paholpolpayuhasena Hospitals are hospitals serving the respective provinces and Sawanpracharak Hospital, is a regional hospital serving an urban population.

### 2.2. Inclusion Criteria for Subjects

The participants were 50 pregnant women aged 20 to 35 years old and their infants who received care at one of the three hospitals. Pregnant women were interviewed during early pregnancy and were selectively recruited at a later visit at 28 weeks gestation if they had reported an intention to breast feed their infants, and to remain in the area after their infant’s birth. Women were excluded if they had: (1) any major health conditions known to adversely affect pregnancy or child outcome (e.g., hypertension, diabetes, and thalassemia), or (2) were being treated with any medications known to interfere with neurodevelopment. Only children born at full term with normal labor were eligible for postnatal follow-up. This study was approved by the Committee on Human Rights Related to Human Experimentation, Faculty of Public Health, Mahidol University and the University of Massachusetts Lowell Institutional Review Board. All of the women provided written informed consent prior to participation in the study.

### 2.3. Interview Information and Urine Sample Collection

Pregnant women served by the three hospitals were recruited from March to December 2011. Medical records of all pregnant women with upcoming appointments at 28 weeks gestation who were receiving prenatal care at one of the three hospitals between March and December of 2011 were evaluated by staff nurses. There were 113 pregnant women who were eligible for follow-up and all 113 agreed to participate in our study. This high participation rate reflects the cultural environment of the national healthcare system, the relationships between the patients and their healthcare professionals, and our ability to conduct the study on the same day as their routine medical appointment. During the appointment that was scheduled around week 28 of gestation, each participant was interviewed by staff to determine general demographic, occupational, and health characteristics, as well as their use of iodine supplementation, and their exposures to pesticides related to home and agricultural activities. Spot urine samples were collected and stored in polyethylene tubes at −45 °C until analysis.

### 2.4. Urine Sample Analysis

Urinary dialkyl phosphates (DAP) were analyzed following the method of Alwis et al. [22]. The following four organophosphate metabolites were assayed: dimethylphosphate (DMP), and three diethylphosphates-diethylphosphate (DEP), diethythiophosphate (DETP), and diethyldithiophosphate (DEDTP). Urine specimens were pretreated using solid-phase extraction and DAP metabolites were reacted with 2,3,4,5,6-pentafluorobenzyl bromide and analyzed by gas chromatography-mass spectrometry (GC-MS). The calibration curves of four dialkyl phosphate (DAP) metabolites were prepared at concentrations of 1, 5, 10, 25, 50, 75, and 100 ng/mL. The average recoveries of the DAP analysis method ranged from 93.64 to 99.92% at DAP concentrations of 10 and 75 ng/mL. The between-day assay coefficients of variation were in the range of 0.59 to 6.45%. The quality control urine samples containing the four DAP metabolites (10 and 75 ng/mL) were analyzed together with urine samples. The detection limits of DMP, DEP, DETP, and DEDTP in urine were 5.000, 0.034, 0.028, and 0.054 ng/mL [4]. Urinary metabolites were expressed as nmol/L, unadjusted for creatinine. Levels in urine samples below the detection limit were recorded as the detection limit divided by two [23]. Total DEP represented the sum of DEP, DETP, and DEDTP, and total DAP was equal to DMP + total DEP. The DMP, total DEP and total DAP levels at seven months of gestation were used to investigate the association of maternal organophosphate pesticide exposure levels with infant scores on the Bayley-III exam.

### 2.5. Neurobehavioral Assessment at Five Months of Age

The Bayley-III [21] was used to assess infant neurobehavioral development at five months of age. Although the Bayley-III consists of five independently administered scales (cognitive, language, motor, social-emotional, and adaptive behavior), we utilized only the cognitive and the motor scales. This decision was based largely on the prior use of the scale in Thailand and the cultural appropriateness of the test items. The cognitive scale assesses mental development using methods that minimize language [24]. Under standardized procedures for administration, the test items evaluate sensorimotor and cognitive development through the observation of the child’s exploration and manipulation of objects, interest in novelty, attention to familiar and unfamiliar stimuli, and problem-solving abilities. The motor scale separately assesses fine motor and gross motor abilities and provides a score for each. The fine motor subtest examines how well children use their eyes, fingers, and hands to explore or manipulate their environment [25]. The gross motor subtest evaluates the child’s ability to control and move their body through observations of head control, limb movement, bodily positioning, locomotion, and coordination.

Two nurses in well-baby clinics from each hospital were trained to administer and score the Bayley according to standardized procedures [21]. This involved a training clinic led by an experienced examiner followed by each trainee’s examination of 10 normal infants, and an evaluation of a videotaped examination to assess the resulting quality of the administration. When needed, additional training was conducted until administration and scoring met acceptable criteria upheld by the trained professional examiner. During the study, Bayley assessments were conducted by the trained nurse examiners when the mothers brought their infants to the well-baby clinic at five months of age. Performance was scored and scaled scores for both the cognitive and motor development indices were determined.

### 2.6. Statistical Analyses

The data were analyzed using SPSS version 18 (SPSS (Thailand) Co., Ltd., Bangkok, Thailand). The difference of urinary organophosphate metabolites found in these pregnant women who did agricultural work and those who did not were tested using Mann–Whitney U test. The relationships between the Bayley scaled scores and demographic data (income of mother, education of mother, testing sites, iodine supplementation, passive smoking, and agricultural occupation of mother) were first examined in univariate analyses to identify significant (*p* ≤ 0.20) demographic covariates. A multiple regression model was then built to examine the association between performance on the Bayley exam (composite cognitive and composite motor scores) and DMP, total DEP, and total DAP metabolite levels, after controlling for the identified significant demographic covariates including education of mother, testing site, iodine supplementation, and agricultural occupation of the mother. All the pregnant women breast feed their babies and due to a change in government policy during the study, all pregnant women who came for prenatal care were supplied with iodine supplements at some point during their pregnancy.

## 3. Results

### 3.1. Recruitment Process

Approximately 150 pregnant women were screened, although some of them were younger than 20 years of age—and so ineligible—and some did not want to participate due to the commitment to use the hospital for all pre and post-natal care. Of the 113 pregnant women who then enrolled in the study, 81 delivered full term infants. The other 32 infants did not qualify for follow-up because 19 were born at other hospitals, 3 were preterm births, and 10 involved birth complications. Of the 81 full term infants who qualified for postnatal follow-up, 19 were lost-to-follow-up due largely to record-breaking, disastrous flooding that occurred in Thailand in 2011. All of the remaining 62 families participated in the Bayley assessments at five months of age (15 from Amnatcharoen Hospital, 16 from Sawanpracharak Hospital, and 31 from Paholpolpayuhasena Hospital). However, due to the loss of 12 urine samples from Sawanpracharak Hospital, only 50 pregnant women are included in this analysis.

### 3.2. Characteristics of Pregnant Women

As shown in Table 1, the average age of the participants was 25.9 years and 40.0% had completed high school or equivalent. In terms of occupation, 50% were farmworkers, 18% were house wives, 4.0% owned a business, and 16.0% were in temporary employment. Only two pregnant women reported drinking alcohol: one drank beer two to four times per week, the other drank beer less than once per month. The income of the families was assessed in a culturally-appropriate manner that involved inquiry about the sufficiency of family income to support family needs, the ability to have savings, and the existence of debt. Most of the pregnant women were classified as earning a sufficient income (88%) as defined by two categories: sufficient with savings (42.0%) and sufficient without savings (46.0%) (Table 1).

At the beginning of this study, the Thai government initiated an iodine supplementation program and all pregnant women who visited prenatal clinics were given iodine supplements (200 µg KI/day) tablets for daily use. Of the pregnant women in this study, 89.8, 96.0, and 96.0% initiated the use of iodine tablet supplements during the first, second, and third trimester of pregnancy, respectively (Table 1). All of the infants had normal Apgar scores at birth. Among the infants, 60% were male. The average age at examination on the Bayley test was 5.91 ± 0.85 months.

### 3.3. Sources of Pesticide Exposure to Organophosphate in 28-Weeks-Pregnant Women

Pregnant women were exposed to organophosphate pesticides both at home and during agricultural work. In 66.0% of the homes, chemical pesticides were used to kill insects (Table 2), and 46% of the pregnant women applied the insecticides at home themselves. Of these, 70% applied insecticides at home once a week. It was common for the women to engage in multiple farming tasks: indeed 18% of the women engaged in three or more tasks. The most common individual tasks done by the women who worked in agriculture were planting and tending the crops (27.7%) and/or hand picking crops, plants, or flowers (28%). With respect to direct application of chemicals in the fields, 12% reported that they applied pesticides and 12.8% reported involvement in controlling weeds with chemicals and hand removal (Table 2). The organophosphate metabolites found in these pregnant women who did agricultural work and those who did not were not significant different by Mann-Whitney U test (*p* > 0.05).

The median of unadjusted urinary DAP levels in samples collected from the pregnant women at week 28 of gestation are shown in Table 3. Levels were 36.83, 15.15, 0.07, and 0.15 nmol/L for DMP, DEP, DETP, and DEDTP, respectively (Table 3). Of the 4 individual metabolites that were measured, DMP was detected most commonly and was presented in 82% of the samples. Concentrations of DMP were also the highest of the four urinary DAP metabolites.

### 3.4. Effects of Pesticide Exposure on Five-Month-Old Infant Neurodevelopment

As shown in Table 4, cognitive composite scores on the Bayley-III for the 50 infants with available data on prenatal metabolite levels ranged from 80 to 120 with an average of 102.1. Motor composite scores ranged from 64 to 136 with an average of 103.12.

Linear regression analyses of the association between organophosphate pesticide exposure (DMP, total DEP, and total DAP) and the Bayley cognitive and motor composite scores were performed with adjustment for the identified significant covariates. The significant covariates included the hospital where testing occurred, the education of the mother, agricultural occupation of mother and iodine tablet supplementation during the first and third trimester of pregnancy.

**Figure 1:**
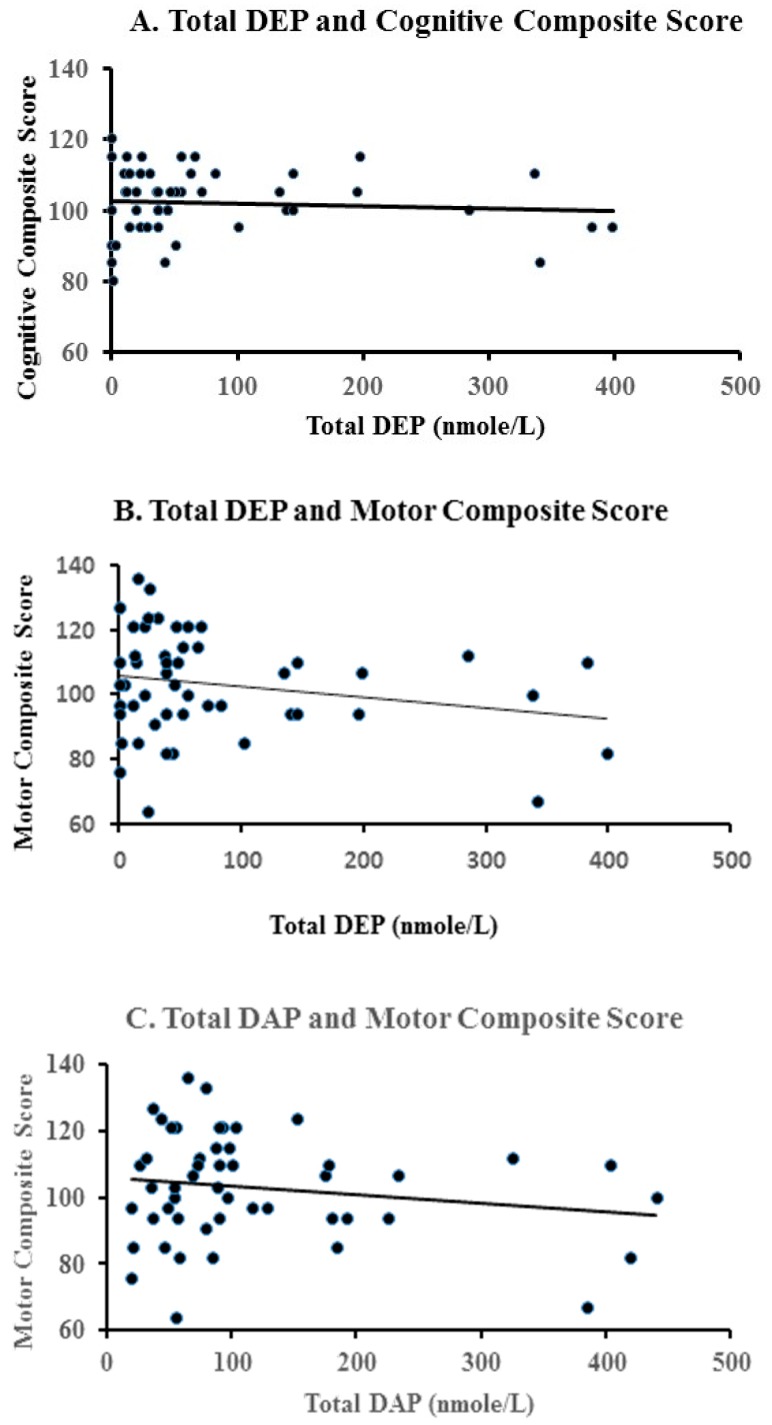
Relationship between prenatal urinary levels of organophosphate pesticide and cognitive and motor composite scores (PDI) on the Bayley-III at five months of age. (**A**) Total DEP and cognitive composite score; (**B**) Total DEP and motor composite score; (**C**) Total DAP and motor composite score.

As shown in Table 5, no significant relationships were found between third trimester DMP levels and Bayley cognitive or motor composite scores. However, higher maternal urinary total DEP levels in the third trimester were associated significantly with reduced Bayley cognitive (*p* = 0.048) and motor composite scores (*p* = 0.025). Similarly, an association between increased total DAP maternal levels at week 28 of gestation and reductions in motor composite scores was significant (*p* = 0.044). The exposure response relationships between these pesticide metabolite levels and the cognitive and motor composite scores are shown in Figure 1.

## 4. Discussion

Our results indicated a significant relationship between prenatal total DEP levels and cognitive and motor performance, and a significant relationship between prenatal total DAP levels and composite motor scores. In the US-based CHAMACOS cohort of women living in an agricultural area in California, no effects on mental or motor Bayley scores were seen at 6 or 12 months of age, however at 24 months of age, declines in mental development scores were associated with increasing prenatal total DAP levels [15]. In the Mount Sinai cohort of pregnant women living in urban housing where pesticides were applied [16], a negative association between increasing total DAP and Bayley Mental Development Index scores at 12 months of age was reported in children born to Black and Hispanic women. Thus, during the first year of life, neither of these cohorts found effects upon motor development as we have found, and the significant effects on mental development were reported at older ages. Differences in cohort demographic characteristics, study designs, and in exposure profiles may account for these differences. In this study, the median level of total DEP (37.80 nmol/L) was higher than that in the CHAMACOS study (22.6 nmol/L) at an average of 25.9 weeks of pregnancy [5]. Also our level of DMP (36.83 nmol/L) versus CHAMACOS (12.0 nmol/L) was higher [5]. The CHAMACOS study measured six DAP metabolites, while we only measured four, but when we added their median DMP and total DEP levels for an estimate of total DAP (34.6 nmol/L) this was lower than our total DAP (85.47 nmol/L). In the CHAMACOS cohort 43% worked in agriculture during their pregnancy and 89% lived with one or more farmworkers. While in our study, 50% of the pregnant women were agriculturists and 68% had family members who worked in agricultural fields. This suggests that exposures to organophosphate pesticides during pregnancy may be higher than in the more regulated US farm.

Among the Mount Sinai inner city cohort, the median total DEP level at an average 31.2 weeks of pregnancy was 24.7 nmol/L compared to our total DEP level of 37.8 nmol/L. They also reported total DMP levels and total DAP levels, but these values included an additional two DMP metabolites we did not measure. As a result, in both the CHAMACOS (rural) and the New York (urban) cohort, the total DMP levels were at least twice that of DEP, whereas, in our sample, the single DMP metabolite and DEP levels were comparable.

It is possible that our detection of this earlier post-natal signal of neurotoxicity may represent a differential response to this higher and possibly different exposure profile. Results at later ages on the children in the CHAMACOS cohort have suggested a delayed impact on the detection of reduced cognitive functioning, with results first seen at two years of age with consistent impact at later ages when multiple domains of functioning have been associated with prenatal exposure [18,19,20]. Likewise, several prospective cohort studies have produced a consistent pattern of early cognitive and behavioral deficits related to prenatal organophosphate exposure in both agricultural and urban populations [15,17,18,19,20]. It has been postulated that a possible explanation for the effects of prenatal organophosphate pesticide exposure is that the organophosphate metabolites can be transferred through placenta to the fetus [5] and, due to immature detoxification pathways, can pose a threat to the unborn child during the rapid brain development.

The limitations of this current study include its small sample size, lack of data on other types of pesticides used, such as herbicides, inability to control for other potential confounders such as diet, exercise, and maternal IQ on neonatal neurodevelopment, or the impact of the disastrous flooding endured by the cohort on the cognitive and motor performance of the infants.

Although the use of a single spot urine sample at 28 weeks of pregnancy to represent prenatal exposure to organophosphate pesticides is a limitation, collection of multiple 24-h urine samples is costly and time consuming to collect. Other researchers in this field have also used one or two spot urine samples to estimate pre-natal exposures for the study of child neurodevelopmental impacts of pesticides [15,16,17,18,19,20]. Unlike adult workplace studies, where creatinine correction of urine samples is common, researchers in the area of prenatal pesticide exposures do not use this method [15,16,17,18,20]. This is due to the rapid physiological changes in pregnant women which result in high intraindividual variability in creatinine excretion [26].

When considering the sources of exposure to organophosphate pesticides for the women in this study, we found that women who performed agricultural activities had slightly higher DAP metabolites than those who did not (*p* > 0.05). This may be because by the 28th week of pregnancy pregnant agriculturists are no longer doing heavy farm labor or working with pesticides. It may be that the most important exposures to organophosphate pesticides could come from pesticide use in the home, eating fruit and vegetables with high OP residues, visiting agricultural fields, or living near agricultural fields where pesticides are sprayed or having farm family members living in the same house that bring home pesticides on their clothes [4]. The half-life of organophosphate pesticides is estimated at 15–30 h, so exposures received through these sources could show up in pre-natal urine samples [27]. If these women continued these exposures post-natally, their infants could continue to be exposed through breast milk since all these breasts fed their infants [28].

## 5. Conclusions

Higher total DEP and total DAP metabolite levels from the mother at week 28 of gestation were significantly associated with reduced motor composite scores on the Bayley-III at five months of age. Total DEP levels were also significantly associated with reduced cognitive composite scores. Additional longitudinal studies are needed to clarify the nature and extent of the neurobehavioral toxicity associated with prenatal exposure to organophosphates as well as to other chemical pesticides. Multiple reports now demonstrate that public health interventions to reduce prenatal pesticide exposures in the rural farming environment, as well as within the home, are needed to prevent long term sequelae from these exposures.

## Figures and Tables

**Table 1:** General characteristics of the mothers and infants (*n* = 50).

Characteristics	Number	Percent
**Maternal**		
Elementary school	11	22.0
Junior high school	20	40.0
Senior high school or vocational certificate	14	28.0
Diploma or higher	5	10.0
**Occupation**		
Farm worker	25	50.0
House wife	9	18.0
Business owner	2	4.0
Temporarily employed	8	16.0
Other occupation	6	12.0
Family member (living within the home) is a farmworker	34	68.0
**Income**		
Sufficient with savings	21	42.0
Sufficient without savings	23	46.0
Not sufficient without debt	4	8.0
Not sufficient with debt	2	4.0
**Iodine Tablet Supplement**		
Iodine supplement at first trimester	45	89.8
Iodine supplement at second trimester	48	96.0
Iodine supplement at third trimester	48	96.0
**Apgar Score at Birth(X; Standard Deviation)**		
1 min after delivery	8.96	1.2
5 min after delivery	9.63	0.6
10 min after delivery	9.80	0.4
**Infant**		
Boy	30	60
Girl	20	40
Age at evaluation (X in months; standard deviation)	5.91	0.85

**Table 2:** Sources of exposure to pesticides and total DAP in 28-weeks-pregnant women (*n* = 50).

Exposure-Related Activities	Yes (%)	Total DAP Median (nmol/L)	No (%)	Total DAP Median (nmol/L)
**At Home**				
Usage of chemical insecticides in the home	66	86.7	33	84.2
Application (by self) of insecticides in the home	46	78.9	54	88.4
**Agricultural Activities or Farm Work**				
Planting and tending of crops	27.7	90.4	72.3	83.1
Application of chemical fertilizer or manure or compost	20	97.6	80	79.2
Application of pesticides	12	93.7	88	83.1
Picking weeds or controlling by chemical use	12.8	147.4	87.2	84.2
Picking crops or plants or flowers	28	91.3	72	79.2
Engagement in three or more agricultural tasks	18	103.1	82	75.5

**Table 3:** Unadjusted urinary DAP metabolite levels (nmol/L) in pregnant women at week 28 of gestation.

Sample Size (*n* = 50)	Detection Frequency (%)	Median	Range
DMP	82.0	36.83	19.83–121.45
Total DEP	88.0	37.80	0.32–399.04
DEP	74.0	15.15	0.11–71.97
DETP	44.0	0.07	0.07–144.45
DEDTP	40.0	0.15	0.14–353.97
Total DAP	96.0	85.47	20.16–440.20

DMP: dimethylphosphate; DEP: diethylphosphate; DETP: diethythiophosphate; DEDTP: diethyldithiophosphate; DAP: dialkylphosphate. Median Total DEP was calculated across the DEP + DETP + DEDTP values for each woman. Median Total DAP was calculated across the DMP + total DEP values for each woman.

**Table 4:** Performance on the Bayley-III at five months of age.

Composite Score	Mean ± Standard Deviation (*n* = 50)	Range
Cognitive (MDI)	102.1 ± 9.32	80–120
Motor (PDI)	103.12 ± 16.02	64–136

**Table 5:** Association between prenatal organophosphate exposure levels and performance on the Bayley-III.

Composite Score	F (1, 49)	β	95% CI	*p*-Value	Change in Composite Score Across IQR Exposure Levels ^b^
**Cognitive (MDI)**					
DMP	2.594	0.108	−0.028, 0.244	0.116	1.514
Total DEP	4.182	−0.024	−0.049, 0.000	0.048 ^a^	−1.769
Total DAP	2.842	−0.020	−0.044, 0.004	0.100	−2.105
**Motor (PDI)**					
DMP	0.943	0.114	−0.123, 0.351	0.338	1.598
Total DEP	5.478	−0.047	−0.088, −0.006	0.025 ^a^	−3.464
Total DAP	4.345	−0.042	−0.082, −0.001	0.044 ^a^	−4.420

DMP: dimethylphosphate; Total DEP: DEP (diethylphosphate) + DETP (diethythiophosphate) + DEDTP (diethyldithiophosphate) values for each woman. Total DAP (dialkylphosphate): DMP + total DEP values for each woman. Metabolite levels were non-creatinine corrected and the analysis adjusted for testing site, maternal education, agricultural occupation of mother and iodine tablet supplementation during the first and third trimesters of pregnancy. ^a^ significant at criterion of *p* ≤ 0.05. ^b^ The change in composite score across IQR of organophosphate exposure levels.
